# Whole-Genome Sequencing Reveals Heterogeneous Resistance Profiles and Selected Mobile Genetic Elements in Ecuadorian Clinical *Enterobacter hormaechei* subsp. *xiangfangensis* and subsp. *hoffmannii*

**DOI:** 10.3390/antibiotics15040387

**Published:** 2026-04-10

**Authors:** Laura Bejarano, Miroslava Anna Šefcová, Karen Muñoz-Mawyin, Isaías Mejía Limones, César Marcelo Larrea-Álvarez, Gabriela Irene Andrade Mena, Erick Saráuz, Pedro Barba, Marco Larrea-Álvarez

**Affiliations:** 1 Carrera de Medicina, Facultad de Ciencias de la Salud, Universidad Espíritu Santo, Samborondón 092301, Ecuador; 2 Department of Materials Science, E.T.S.I. Caminos, Universidad Politécnica de Madrid, C/Profesor Aranguren 3, 28040 Madrid, Spain; 3 Laboratorio Clínico del Hospital General San Vicente de Paúl, Ibarra 100105, Ecuador; 4 Carrera de Biotecnología, Facultad de Ingeniería en Ciencias Agropecuarias y Ambientales, Universidad Técnica del Norte, Ibarra 100105, Ecuador

**Keywords:** *Enterobacter hormaechei* subsp. *xiangfangensis*, *Enterobacter hormaechei* subsp. *hoffmannii*, multidrug resistance, whole-genome sequencing, integrons, Ecuador

## Abstract

**Background:** *Enterobacter hormaechei*, a member of the *Enterobacter cloacae* complex (ECC), is increasingly recognized as a multidrug-resistant (MDR) nosocomial pathogen. However, subspecies-level genomic data from Ecuador remain limited. **Methods:** Four clinical *E. hormaechei* isolates from a hospital in northern Ecuador were analyzed using antimicrobial susceptibility testing and whole-genome sequencing (WGS). Genomic characterization included multilocus sequence typing (MLST), resistome profiling, plasmid replicon detection, integron screening, genomic island analysis, and phylogenetic comparison with publicly available Ecuadorian genomes. **Results:** WGS identified three isolates as subsp. *xiangfangensis* (ST136 and ST337) and one as subsp. *hoffmannii* (ST145). Two ST136 isolates exhibited extensive MDR phenotypes associated with *bla*_CTX-M-15_, *bla*_OXA-1_, *bla*_ACT-16_, and additional aminoglycoside and fluoroquinolone resistance genes. ST145 showed moderate resistance, whereas ST337 remained largely susceptible despite harboring *bla*_ACT-16_. Multiple genomic islands and plasmid replicons (IncF/IncR or IncHI2) were detected. Phylogenetic analysis demonstrated clustering with previously reported Ecuadorian lineages. **Conclusions:** This study provides subspecies-level genomic characterization of clinical *E. hormaechei* in Ecuador and describes heterogeneous resistance profiles and associated mobile genetic elements, contributing baseline data for regional surveillance.

## 1. Introduction

The genus *Enterobacter* occupies diverse niches as an environmental saprophyte, plant endophyte, gut commensal, and bioprocessing agent; however, specific lineages emerge as opportunistic pathogens within the ESKAPE (*Enterococcus faecium*, *Staphylococcus aureus*, *Klebsiella pneumoniae*, *Acinetobacter baumannii*, *Pseudomonas aeruginosa*, and *Enterobacter* species) group, associated with nosocomial outbreaks [1,2]. Among these, the *Enterobacter cloacae* complex (ECC), a diverse group comprising several species such as the epidemiologically relevant *E. hormaechei*, *E. cloacae*, *E. kobei*, and *E. ludwigii*, is most frequently encountered in clinical settings [3]. These species are recognized for their intrinsic and acquired mechanisms of antimicrobial resistance (AMR), which complicates treatment. ECC species possess intrinsic AmpC-mediated resistance [3] and are increasingly reported as nosocomial pathogens exhibiting multidrug resistance due to the acquisition of ESBLs (e.g., TEM, SHV, CTX-M) [4] and carbapenemases (e.g., KPC, NDM, IMP/VIM) [5].

Species-level identification within the complex remains challenging with conventional microbiological methods, as they cannot reliably differentiate between its constituent species [3]. Similarly, matrix-assisted laser desorption/ionization time-of-flight mass spectrometry (MALDI-TOF MS), while rapid, frequently fails to provide resolution at the subspecies level in some cases [6,7]. This limitation hinders the accurate tracking of individual subspecies, which may exhibit distinct resistance patterns and outbreak potential. Consequently, definitive identification requires molecular techniques, primarily whole-genome analysis [8]. *E. hormaechei* is recognized as a causative agent of various infections such as bacteremia, pneumonia, urinary tract infections (UTIs), and wound/surgical site infections [7,8]. This species comprises subspecies associated with nosocomial outbreaks, particularly in intensive care units (ICUs), including *E. hormaechei* subsp. *hormaechei*, subsp. *steigerwaltii*, subsp. *hoffmannii*, and subsp. *xiangfangensis* [9,10,11,12,13]. The ability of *E. hormaechei* to acquire and disseminate resistance traits to, for instance, broad-spectrum β-lactams and carbapenems, as well as virulence factors, has been well documented [14,15,16,17,18]. This genomic plasticity supports the evolution of these pathogens and may limit available therapeutic options.

WGS has become essential for resolving the taxonomy of the ECC, investigating hospital outbreaks, and comprehensively profiling resistance and virulence determinants in *E. hormaechei* [7]. Genomic profiling reveals an extensive array of horizontally acquired antimicrobial resistance genes. These include prevalent plasmid-borne β-lactamases, such as extended-spectrum variants (CTX-M, TEM, SHV), AmpC enzymes (ACT, CMY, DHA), and carbapenemases (NDM, KPC, OXA-48-like), alongside genes conferring resistance to aminoglycosides (*aac*, *aph*), fluoroquinolones (*qnr*), fosfomycin (*fosA*), sulfonamides, trimethoprim, and tetracyclines [9,11,12,13,15,16,19,20]. Pathogenesis is supported by critical virulence determinants, notably high-affinity iron-acquisition systems (enterobactin, aerobactin), a functional Type VI Secretion System (T6SS) for interbacterial competition, and adhesins involved in biofilm formation (curli fibers, cellulose) [9,15,19,20]. These adaptive traits can be disseminated by mobile genetic elements, primarily conjugative plasmids of the IncF, IncHI2, and IncX3 families [9,11,15,19,20], which are commonly associated with class 1 integrons. Furthermore, genomic islands integrate larger cassettes of genes responsible for antibiotic resistance, heavy metal resistance (*ars*, *pco*, *sil*), and niche-specific virulence, which collectively drive rapid adaptation and persistence in diverse settings [20].

Multilocus sequence typing (MLST) is crucial for tracking the local and international spread of successful lineages. The global spread of multidrug-resistant *E. hormaechei* is largely attributed to various successful lineages. Among these, the KPC-associated ST78, the NDM-disseminating ST114 (a member of CC114), the widely reported ST66 (often identified as subspecies *xiangfangensis*), and the European VIM-linked ST90 represent widely reported and clinically relevant sequence types [15,21,22,23]. In addition to the major epidemic clones, surveillance studies consistently report a range of other clinically significant sequence types (STs). Among these, ST145 and ST45 are frequently linked to the spread of carbapenemases. Other prevalent types highlight distinct epidemiological patterns. For instance, ST133 has been associated with bloodstream infections, while ST171 and ST93 represent emerging *bla*_NDM-5_-carrying lineages. Together, these types illustrate the international distribution, genetic diversity, and niche adaptation of multiple *E. hormaechei* lineages, contributing to the complex global epidemiology of this pathogen [5,18,24,25,26,27].

Strains of *E. hormaechei* have been documented in countries within the Andean region, including members of the Andean Community (Bolivia, Colombia, Ecuador, and Peru) and the former member state, Venezuela. In Colombia, MDR isolates from guinea pigs carried carbapenemase genes, including *bla*_KPC_ and *bla*_OXA-48_ [28], while other lineages were identified in landfill leachates [29]. Similarly, a fatal clinical case in Venezuela involved an MDR *E. hormaechei* subsp. *hormaechei* strain co-harboring *bla*_KPC_ and *bla*_VIM_, exhibiting resistance to nearly all antibiotic classes tested [30]. In Peru, clinical isolates of the high-risk clone ST2054 have been reported, carrying the *bla*_NDM-1_ gene [31]. Bolivia has documented clinical isolates harboring the plasmid-borne colistin resistance gene (*mcr-1*) on an IncI2 plasmid [32]. Finally, genomic surveillance in Ecuador has identified a diverse array of resistant *Enterobacter* species, including *E. kobei* ST2070 co-harboring *bla*_KPC-2_ and *bla*_OXA-10_ [33], *E. asburiae* carrying *mcr* genes [34], and *E. cloacae* A1 ST1608 [35]. However, the current genomic characterization of *E. hormaechei* primarily confirms its presence as a multidrug-resistant, ESBL-producing pathogen [36]. A significant limitation remains the absence of standardized MLST data, as sequence types (STs) are frequently unreported, limiting detailed clonal tracking. Therefore, determining the presence and evolutionary relationships of clonal lineages in this region requires genomic surveillance that includes circulating sequence types, detailed gene carriage, and phylogenetic analysis.

This study aimed to characterize four clinical *E. hormaechei* isolates from a hospital in Imbabura, Ecuador, using combined genomic and phenotypic approaches. In the Ecuadorian context, this work provides the first subspecies-level genomic resolution of *E. hormaechei* and identifies multiple sequence types, including ST136, ST145, and a novel ST related to ST337. It also registers mobile genetic elements (integrons, plasmids, and genomic islands) and documents heterogeneous carbapenem susceptibility phenotypes in the absence of detectable carbapenemase genes. Together, these findings provide baseline insight into the diversity and resistance architecture of this species in an understudied setting.

## 2. Results

Four revived clinical isolates were identified as *E. hormaechei* by MALDI-TOF MS. Susceptibility testing confirmed moderate to multidrug-resistant phenotypes (Table 1), while WGS distinguished the wound isolates as *E. hormaechei* subsp. *xiangfangensis* and the urinary isolate as subsp. *hoffmannii*, while also revealing key resistance, virulence, and mobile genetic determinants (Table 2; Supplementary Results, Raw data).

### 2.1. Isolate Susceptibility and Resistance Genes

ENH_002 exhibited resistance primarily to early-generation β-lactams (ampicillin, amoxicillin/clavulanate, piperacillin/tazobactam, and cefuroxime), a pattern consistent with the presence of the *bla*_ACT-14_ gene encoding an AmpC-type β-lactamase. Its susceptibility to later-generation β-lactams, aminoglycosides, and quinolones aligns with the absence of additional resistance determinants. The detected *aadA1* gene confers resistance only to streptomycin and spectinomycin, which were not tested. Although the sulfonamide resistance gene *sul1* was identified, phenotypic resistance to co-trimoxazole was not observed, which may reflect the absence of trimethoprim resistance genes (*dfr*), as *sul1* alone is insufficient to confer co-trimoxazole resistance. ENH_003 showed a broader β-lactam resistance pattern, including third-generation cephalosporins and aztreonam, which is consistent with the presence of the *bla*_ACT-16_ AmpC variant. The isolate remained susceptible to cefepime, meropenem, and imipenem, while exhibiting resistance to ertapenem. No carbapenemase genes were detected. Analysis of OmpC revealed synonymous substitutions and several nonsynonymous mutations resulting in amino acid changes (A177P, I211L, A222D, A227M, Y228A, and N231E). In contrast, OmpF showed a reduced protein length of 352 amino acids compared with 363 amino acids in the reference sequence, accompanied by amino acid changes at positions 48, 49, and 50 (Supplementary Results, Figure S1). The functional impact of these alterations was not assessed. Resistance to tetracycline and intermediate resistance to chloramphenicol are consistent with the presence of *tet(B)* and *catA1*, respectively. Intermediate susceptibility to tobramycin and gentamicin, in the absence of aminoglycoside-modifying enzyme genes, suggests possible permeability or efflux-mediated mechanisms.

Isolates ENH_004 and ENH_007 exhibited an extensive MDR phenotype, demonstrating resistance to nearly all major antibiotic classes tested (Table 1). This broad resistance profile correlates with a complex and nearly identical set of 15 core resistance genes, consistent with close genetic relatedness between the two isolates (Table 2). Resistance to β-lactams is consistent with the presence of multiple β-lactamase genes. The ESBL gene *bla*_CTX-M-15_ is associated with resistance to extended-spectrum cephalosporins, while *bla*_ACT-16_ may further contribute to reduced susceptibility. The additional β-lactamases *bla*_TEM-1B_ and *bla*_OXA-1_ likely contribute to penicillin resistance. Differential susceptibility to cefepime and ceftazidime between isolates may reflect additional mechanisms not evaluated here. Non-susceptibility to ertapenem and meropenem (in ENH_007) was observed based on disk diffusion, despite the absence of carbapenemase genes; however, MIC testing would be required to more precisely define the carbapenem resistance phenotype. Both isolates exhibited OmpC coding sequence characteristics similar to those observed in ENH_003, whereas OmpF showed a marked truncation, with a reduced length of 222 amino acids compared with 363 amino acids in the reference sequence (Supplementary Results, Figure S1). The functional consequences of these alterations on porin activity or membrane permeability were not determined. The *aac(3)-IIa* gene correlates with resistance to tobramycin and gentamicin. The intermediate (ENH_004) or resistant (ENH_007) phenotype to amikacin may reflect the combined activity of aminoglycoside-modifying enzymes, although additional mechanisms cannot be excluded. The dual-function gene *aac(6′)-Ib-cr* is associated with reduced susceptibility to both aminoglycosides and fluoroquinolones. Resistance to tetracycline and co-trimoxazole is consistent with *tet(A)* and the *dfrA14*/*sul2* pair, respectively. Although *catB3* was detected, chloramphenicol susceptibility was observed, suggesting possible differences in gene expression or regulation. Minor phenotypic divergences between the isolates may reflect regulatory or permeability differences not assessed in this study. The *fosA* gene—encoding a glutathione S-transferase that inactivates fosfomycin—was identified in isolates ENH_002, ENH_004, and ENH_007 (Table 2). Phenotypic susceptibility to fosfomycin was not determined due to the lack of validated disk diffusion methods for this genus and the unavailability of MIC testing in this study. Consequently, the clinical relevance of this genetic finding remains undefined, although the presence of this gene could be considered a predictor of resistance.

### 2.2. Virulence Factors and Environmental Persistence Traits

All isolates shared several genes associated with host interaction, including factors involved in regulation, immune evasion, adhesion/biofilm formation, and invasion. The enterobactin-mediated iron acquisition system was present in all genomes (Table 2). Components of the Type VI Secretion System (T6SS) gene cluster were identified in isolates ENH_004 and ENH_007, whereas ENH_002 carried a single component and ENH_003 lacked detectable T6SS-associated genes. Differences were also observed in environmental resistance gene content (Table 2). ENH_002 carried the most extensive set of environmental resistance determinants, including a complete Locus of Heat Resistance (LHR) cluster containing genes such as *clpK*, *hsp20*, *shsP*, *trxLHR*, *psi-GI*, *yfdX1/X2*, and *hdeD-GI*, which have been associated with enhanced tolerance to elevated temperatures. This isolate also harbored complete copper (*pcoABCDERS*), silver (*silABCEFPRS*), and tellurium (*terDWZ*) resistance operons, linked to tolerance to heavy metal exposure. In contrast, ENH_003 possessed only tellurium resistance genes (*terDWZ*) and a single arsenate reductase (*arsC*), with no LHR-associated genes detected. Isolates ENH_004 and ENH_007 shared identical heavy metal resistance profiles, each carrying copper and silver resistance operons but lacking the LHR cluster and tellurium resistance genes identified in ENH_002.

### 2.3. Integrons, Plasmids, and Genomic Islands

ENH_002 carried the core structural components of a class 1 integron, including the integrase gene (*intI1*), the primary recombination site (*attI*), and the 3′ conserved segment containing the truncated *qacEΔ1* gene. This configuration is consistent with a class 1 integron structure capable of capturing gene cassettes. The *aadA4* and *sul1* genes were identified in the same contig as these sequences and near an IncR replicon. In addition, this isolate carried multiple plasmid replicons, including IncFIA(HI1), IncFIB(pECLA), and IncFII(pECLA) (Table 2; Supplementary Results, Figure S2). In contrast, isolates ENH_004 and ENH_007 were positive only for *intI*, which may reflect incomplete or truncated integron structures. The *dfr* genes were identified in the same contig as *intI* in the draft assemblies; however, no plasmid replicons were detected in these isolates. While this may suggest a chromosomal location of these determinants, complete plasmid reconstruction would be required for confirmation. ENH_003 carried IncHI2 and IncHI2A replicons, and *catA1* was identified near plasmid-associated sequences, although integron structures were not detected in this isolate (Table 2; Supplementary Results, Figure S2).

**Figure 1:**
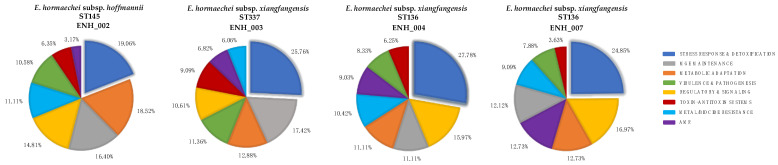
Functional distribution of genes within genomic islands across four *E. hormaechei* isolates. Pie charts show the relative percentage of genes assigned to each functional category. MGE: mobile genetic element; AMR: antimicrobial resistance.

All four *E. hormaechei* isolates harbored multiple genomic islands (GIs), consistent with horizontal gene acquisition (Table 2). ENH_002 carried 42 GIs (198 genes), ENH_003 had 45 (132 genes), ENH_004 contained 34 (144 genes), and ENH_007 possessed 36 (165 genes). Across isolates, stress response and detoxification genes represented the most abundant functional category within GIs. Despite this shared pattern, differences in functional composition were observed. ENH_002 showed a relatively balanced GI profile, with notable representation of metabolic adaptation genes (17.7%) and metal/biocide resistance determinants (10.6%), including the copper (*pco*) and silver (*sil*) operons, as well as the virulence-associated gene *ompA*. ENH_003 exhibited a higher proportion of toxin–antitoxin systems (9.1%) and mobile genetic element-related functions (17.4%), and its islands included arsenic resistance (*ars*) and antimicrobial resistance genes (*tet*, *cat*). In contrast, ENH_004 and ENH_007 showed greater representation of regulatory and signaling functions (16% and 17%, respectively) and antimicrobial resistance genes (9% and 12.7%), including *fosA*, *aac*, *bla_TEM/OXA/CTX-M_*, *tet*, *cat*, *dfr*, and *sul*, along with the *pco* and *sil* operons (Figure 1). Putative prophage regions and CRISPR-Cas elements are provided in the Supplementary Results. Prophages and CRISPR Cas. These analyses were performed on draft genomes derived from short-read sequencing, which limits the accurate resolution of prophage boundaries, integration sites, and CRISPR array architecture. Accordingly, these outcomes should be considered preliminary and were not further validated.

### 2.4. Phylogenetic Tree

**Figure 2:**
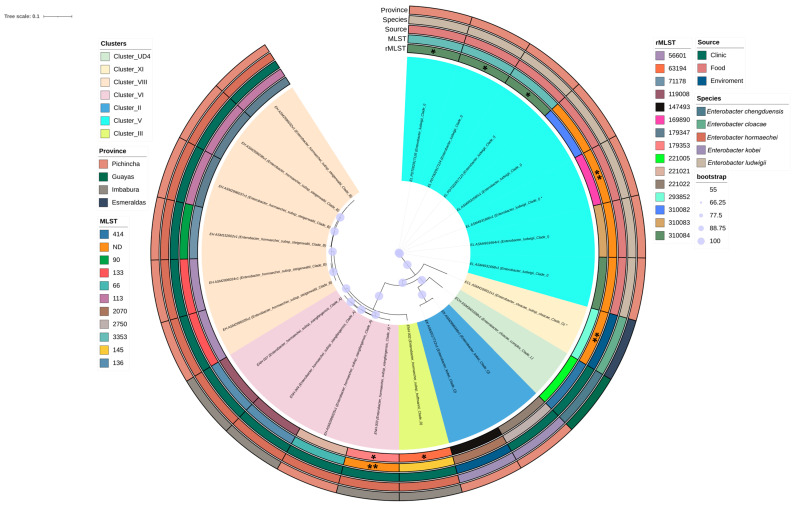
Phylogenetic tree based on core genome genes from 22 *Enterobacter* isolates reported in Ecuador. Branch support is indicated by inner circles representing ultrafast bootstrap values, ranging from 55 to 100. Background colors denote the genomic clusters assigned to each isolate. Clade assignments correspond to species/subspecies as follows: Clade A, *E. hormaechei* subsp. *xiangfangensis*; Clade B, *E. hormaechei* subsp. *steigerwaltii*; Clade D, *E. hormaechei* subsp. *hoffmannii*; Clade Q, *Enterobacter kobei*; Clade I, *Enterobacter ludwigii*; Clade L, *E. cloacae* complex; Clade G, *E. cloacae* subsp. *cloacae*. The metadata tracks show, from the inner to outer ring, ribosomal sequence type (rST), sequence type (ST), source, species, and province of origin. * Asterisks adjacent to species names denote isolates harboring novel sequence types (STs). These novel STs are additionally indicated in the ST track by double asterisks (**). In the rST track, isolates without a definitive assignment were categorized according to the closest related group and are indicated by a single asterisk (*).; ENH_003 represents a novel ST most closely related to ST337.

MLST-based phylogenetic analysis of 22 *Enterobacter* genomes (four clinical isolates from this study and 18 publicly available genomes from Ecuador) clustered the population into seven clades consistent with species and subspecies designations (Figure 2). The four isolates from this study were distributed among three clades. Three isolates (ENH_003, ENH_004, ENH_007) clustered within Clade A, corresponding to *E. hormaechei* subsp. *xiangfangensis*. Within this clade, the two ST136 isolates (ENH_004 and ENH_007) formed a monophyletic group and were phylogenetically close to a clinical ST66 isolate from Pichincha, suggesting relatedness to strains circulating in northern Ecuador. The third isolate, ENH_003, represented a novel sequence type most closely related to ST337 and branched separately within the same clade, indicating a distinct but related lineage. In contrast, the urinary isolate ENH_002 (ST145) clustered in Clade D, corresponding to *E. hormaechei* subsp. *hoffmannii*, and was phylogenetically distinct from the *xiangfangensis* isolates.

The remaining clades corresponded to other Enterobacter lineages and were associated with distinct ecological sources. Clade B (*E. hormaechei* subsp. *steigerwaltii*) formed a sister group to Clade A. Other clades included *Enterobacter kobei* (Clade Q; clinical and environmental isolates from Pichincha), *Enterobacter ludwigii* (Clade I; food chain-associated isolates from Pichincha), *E. cloacae* complex (Clade L; clinical isolates from Guayas), and *E. cloacae* subsp. *cloacae* (Clade G; environmental isolates from Esmeraldas). In general, this phylogeny indicates that *Enterobacter* populations in Ecuador comprise multiple genetically distinct lineages associated with diverse ecological sources. The available data suggest considerable diversity within Pichincha, although broader geographic sampling is required to fully resolve national distribution patterns.

## 3. Discussion

This genomic analysis provides, to our knowledge, the first subspecies-level genomic description of *E. hormaechei* subsp. *xiangfangensis* and *hoffmannii* from Ecuador. The isolates exhibited moderate to multidrug resistance, consistent with the presence of genes such as *bla*_CTX-M-15_ and *bla*_OXA-1_. Phylogenetic analysis placed the isolates into distinct clades. Wound-associated *xiangfangensis* strains clustered with a previously reported regional clinical isolate, supporting the presence of related lineages circulating locally.

*E. hormaechei* subsp. *xiangfangensis and steigerwaltii* are commonly reported among clinical isolates [9,12,13,37]. Consistent with this, MLST analysis showed that three of our four isolates clustered within subsp. *xiangfangensis* (Clade A), together with ST66, reported in Pichincha, indicating related lineages in the region. The two *xiangfangensis* ST136 isolates (ENH_004 and ENH_007) formed a monophyletic group and exhibited highly conserved core genomes, resistomes, and virulence profiles. They differed mainly in genomic island number. This pattern is consistent with clonal expansion from a common ancestor, with diversification likely driven by recombination and horizontal gene transfer rather than deep phylogenetic separation [38,39]. ST136 is rarely documented and inconsistently classified within the ECC. It has been identified in humans and companion animals as *E. hormaechei* subsp. *xiangfangensis* [40,41]. In contrast, reports from nosocomial infections in Canada, Lebanon, and Tunisia describe it only as *E. hormaechei* [42,43,44], and a canine isolate in Japan as *E. cloacae* [45]. In this study, its detection in surgical, traumatology, and ICU respiratory isolates documents its occurrence in diverse clinical contexts. ST66 is recognized as a high-risk clone of *xiangfangensis*, as it has been linked to multiple reports in clinical settings [23,24,40]. Moreover, ENH_003 represented a novel sequence type most closely related to ST337, highlighting additional diversity within this subspecies. The absence of this ST from the published literature suggests it may represent a rare or previously underreported lineage.

The *E. hormaechei* subsp. *steigerwaltii* isolates described from clinical samples in Pichincha formed a distinct clade (Clade B) comprising ST113, ST90, and ST133. These lineages have been documented in association with human infection, although their ecological distributions differ. ST133 and ST90 have been frequently identified in clinical infections [46,47,48,49], whereas ST113 shows a broader ecological range, including hospital-adjacent environments, human samples, and veterinary settings such as horses and animal clinics [50,51,52,53]. *E. hormaechei* subsp. *hoffmanniii* is also commonly reported from clinical settings [18,54,55]. The distinct placement of subsp. *hoffmannii* ST145 (ENH_002) supports subspecies-level differentiation within the phylogeny. ST145 is infrequently reported and has previously been identified in clinical isolates from France, Chile, and Poland [18,24,56]. Its detection here in a community-acquired urinary isolate documents its occurrence outside hospital-associated settings. Overall, the phylogeny indicates the presence of multiple related and less common *E. hormaechei* sequence types co-occurring within Ecuador, a pattern that reflects both its genetic diversity and its recognized capacity to acquire resistance to multiple antimicrobial classes [1,57].

The *E. hormaechei* subsp. *hoffmannii* ST145 isolate analyzed here exhibited a moderate resistance profile, primarily associated with the AmpC β-lactamase gene *bla*_ACT-14_ and accompanied by *aadA4*, *sul1*, and *fosA*. This profile resembles the limited resistance reported for a clinical ST145 isolate from Chile [18]. In contrast, extensively MDR ST145 strains described in clinical and animal sources in Europe harbor broader resistance repertoires. These include carbapenemases (*bla*_VIM_), ESBLs (*bla*_CTX-M-15_, *bla*_SHV-12_), quinolone resistance determinants (*qnr*, *aac(6′)-Ib-cr*), and multiple aminoglycoside-modifying enzymes [24,50,56]. Collectively, available data suggest that ST145 encompasses a heterogeneous resistance spectrum, ranging from limited to extensive profiles across different clinical contexts.

The MDR *E. hormaechei* subsp. *xiangfangensis* ST136 isolates were characterized by the co-occurrence of ESBL and AmpC genes, together with determinants associated with resistance to aminoglycosides, quinolones, tetracycline, chloramphenicol, folate antagonists, and fosfomycin. The close agreement between resistome composition and phenotypic profiles is consistent with a shared evolutionary background. Minor differences may reflect regulatory or expression-level variation. This resistance profile is comparable to those reported for ST136, a lineage documented in hospital settings and companion animals [40,58]. Previous reports indicate that ST136 isolates share a conserved genetic core, including *bla*_CTX-M-15_, *bla*_ACT_ variants, and aminoglycoside-modifying enzymes [41,43]. The co-occurrence of *bla*_CTX-M-15_ and *bla*_OXA-1_, together with *fosA*, may restrict certain therapeutic options in Enterobacterales infections [59,60]. However, variation in the presence of high-impact carbapenemases (e.g., *bla*_NDM_, *bla*_OXA-48_) has been reported and appears to influence the overall resistance phenotype [41,43].

The ST136 isolates of subsp. *xiangfangensis*, carrying multiple *bla* genes, showed carbapenem non-susceptibility based on disk diffusion. This included resistance to ertapenem and reduced susceptibility to meropenem, with one isolate reaching full resistance. However, the absence of MIC or confirmatory phenotypic testing limits precise classification of these phenotypes, particularly for borderline susceptibility. MIC determination would be valuable to better define the resistance profile. In the absence of acquired carbapenemase genes, this phenotype may reflect the combined effect of ESBL and AmpC production. This effect may be enhanced by reduced outer membrane permeability, a mechanism commonly reported and associated with early loss of ertapenem susceptibility [61,62,63]. The OmpC and OmpF alterations observed in this study, including truncations in OmpF, may be consistent with this mechanism. However, their functional impact was not assessed and should be considered hypothetical. In contrast, the *xiangfangensis* ST337-related isolate, harboring only *bla*_ACT-16_, exhibited an overall susceptible profile. Resistance was limited to ertapenem and preserved susceptibility to meropenem and imipenem, a pattern that may also involve limited permeability changes in addition to AmpC activity [61,62,63]. This profile is comparable to low-resistance *xiangfangensis* clinical isolates reported from China and South Korea, such as ST527, which harbor a limited set of resistance determinants (e.g., *bla*_ACT-16_ and *fosA*) [64,65]. Altogether, the detection of an MDR lineage (ST136), a variably resistant lineage (ST145), and a largely susceptible lineage (ST337) illustrates the heterogeneity of resistance phenotypes within *E. hormaechei*, which may complicate empirical therapeutic decision-making.

All isolates in this study retained a conserved repertoire of virulence-associated genes, including determinants involved in adhesion and biofilm formation (*csgG*), outer membrane structure and invasion (*ompA*), global regulation (*rpoS*, *phoP*, *rcsB*, *fur*), and enterobactin-mediated iron acquisition (*ent*/*fep*) [20,66,67,68]. These factors are consistent with traits associated with colonization and environmental persistence [69,70]. Type VI Secretion System (T6SS) components were detected only in the MDR isolates (ST136), suggesting potential lineage-associated differences in interbacterial competition and niche adaptation [71]. Consistent with previous reports, variation in virulence gene content was observed between lineages, with subsp. *hoffmannii* ST145 encoding *mrkA*, *nlpI*, and *terC*, and the *xiangfangensis* ST136 clade characterized by *nlpI* and *terC* [50]. All analyzed isolates carried heavy metal resistance determinants, indicating a shared genetic capacity for environmental tolerance under selective pressures such as those encountered in healthcare settings [72,73]. Notably, *E. hormaechei* subsp. *hoffmannii* encoded additional niche-associated traits, including a complete Locus of Heat Resistance and multiple metal resistance operons, suggesting an increased potential for tolerance to thermal stress and disinfectant exposure [74]. The coexistence of these persistence-associated traits may contribute to the ecological success of *E. hormaechei* across clinical and environmental contexts [20,75].

A structurally complete class 1 integron was identified in *E. hormaechei* subsp. *hoffmannii*, associated with the *aadA4* and *sul1* genes, consistent with the capacity to acquire resistance cassettes [76]. In contrast, only the integrase gene was detected in *E. hormaechei* subsp. *xiangfangensis* isolates, which may indicate incomplete or remnant integron structures. Class 1 integrons have been reported in the *xiangfangensis* subspecies, with some located on conjugative plasmids [9,54,77], although no plasmid replicons were detected in ST136 in this study. In contrast, the ST337-related isolate carried IncHI2 replicons, with the *catA1* gene located in the same contig; these plasmids are known to carry multiple resistance determinants in Enterobacteriaceae [78,79] and in *E. hormaechei* [50,66,80]. In the ST145 isolate, the proximity of the class 1 integron to the IncR replicon suggests a plasmid-associated location [81,82,83,84,85]. This isolate also harbored a multi-replicon IncF plasmid (IncFIA, IncFIB, IncFII), which is associated with an enhanced capacity for gene acquisition due to its size and composite genetic content in this subspecies [13,68]. All analyzed genomes carried numerous genomic islands enriched in stress response and detoxification functions, consistent with roles in environmental tolerance [20,72,86]. Differences in functional composition were observed between lineages: subsp. *hoffmannii* showed enrichment in metabolic and heavy metal/biocide resistance genes, whereas subsp. *xiangfangensis* exhibited a higher relative abundance of antimicrobial resistance genes.

This study has several limitations, including the small number of isolates, its single-center design, and its limited temporal coverage (March 2021), which restrict the generalizability of the findings. In addition, detailed clinical metadata were not available, as isolates were analyzed without access to patient records; therefore, information on clinical outcomes, prior antibiotic exposure, and hospitalization history could not be assessed, limiting interpretation of clinical impact. Reliance on short-read sequencing further limits complete plasmid reconstruction and detailed characterization of complex mobile genetic elements, and the absence of phenotypic validation for fosfomycin resistance and environmental stress tolerance constrains functional interpretation. Accordingly, our results should be interpreted as providing baseline genomic and phylogenetic insights rather than representing national epidemiology. Within these constraints, this analysis offers a subspecies-level genomic description of *E. hormaechei* subsp. *xiangfangensis* and *hoffmannii* from Ecuador and documents the coexistence of MDR and susceptible lineages in a single healthcare setting. Further studies with broader sampling and integrated clinical data will be necessary to better define resistance patterns and lineage diversity within the ECC in Ecuador.

## 4. Materials and Methods

### 4.1. Bacterial Isolates

The four isolates analyzed in this study were obtained from the bacterial culture collection of the clinical microbiology laboratory at Hospital San Vicente de Paúl, Ibarra, Ecuador, following routine diagnostic workup in March 2021. Original laboratory identifiers (AH_/AO_) were reassigned to standardized codes (ENH_) for consistency throughout the study; both designations are provided here for reference. These isolates were provided exclusively for microbiological characterization. Available information was limited to sample type and general clinical context (e.g., ICU vs. community), with no patient-identifying information, clinical outcomes, prior antibiotic exposure, or hospitalization details. No human subjects or identifiable data were involved. Isolates were cryopreserved at −80 °C for subsequent characterization. The isolates originated from distinct clinical sources: AH_003 (surgical wound), AH_004 (traumatology wound), AH_007 (respiratory sample, intensive care unit), and AO_002 (community-acquired urine). The cryopreserved isolates were revived at 37 °C for 24 h on Eosin Methylene Blue (EMB) agar (Oxoid, Basingstoke, Hampshire, UK), a medium selected for its selectivity against Gram-positive bacteria and its capacity for preliminary differentiation within the Enterobacteriaceae family. Following observation of mucoid, purple colonies on EMB agar, isolates were subjected to standard biochemical profiling. The test panel comprised triple sugar iron (TSI) agar, Simmons citrate agar, lysine iron agar (LIA) (Oxoid, Basingstoke, Hampshire, UK), motility-indole-ornithine (MIO) medium, and urea broth (Becton-Dickinson, Franklin Lakes, NJ, USA) [87]. Biochemical profiling yielded results characteristic of *Enterobacter* spp. All isolates were citrate-positive, urease-negative, with an acid/acid reaction and gas production on TSI agar (no H_2_S). LIA showed a purple butt (lysine decarboxylase-positive), and the MIO test was positive for motility and ornithine decarboxylase but negative for indole [87]. Because biochemical profiling cannot reliably differentiate species within the *Enterobacter cloacae* complex, definitive identification was obtained via MALDI-TOF (Bruker Daltonik GmbH, Bremen, Germany). All isolates were identified as *E. hormaechei* and subsequently designated as ENH_003 (AH_003; surgical wound), ENH_004 (AH_004; traumatology wound), ENH_007 (AH_007; respiratory sample from ICU), and ENH_002 (AO_002; community-acquired urine).

### 4.2. Antimicrobial Susceptibility Testing

All *E. hormaechei* isolates were tested for antimicrobial susceptibility using the disk diffusion method (Kirby–Bauer) on Mueller–Hinton agar (Oxoid, Basingstoke, Hampshire, UK) with antibiotic disks (Oxoid, Basingstoke, Hampshire, UK) of the following drugs: ampicillin (10 µg), amoxicillin/clavulanate (20/10 µg), piperacillin/tazobactam (100/10 µg), cefuroxime (30 µg), cefotaxime (30 µg), ceftazidime (30 µg), cefepime (30 µg), aztreonam (30 µg), ertapenem (10 µg), meropenem (10 µg), imipenem (10 µg), tobramycin (10 µg), gentamicin (10 µg), amikacin (30 µg), ciprofloxacin (5 µg), levofloxacin (5 µg), tetracycline (30 µg), chloramphenicol (30 µg), and co-trimoxazole (trimethoprim–sulfamethoxazole, 1.25/23.75 µg). The *E. coli* ATCC 25922 strain was used as quality control. Categories (resistant, intermediate, and susceptible) were interpreted according to the Clinical and Laboratory Standards Institute guidelines (CLSI M100-Ed35, 2025) [88].

### 4.3. Whole-Genome Sequencing (WGS)

The QIAamp UCP Pathogen Mini Kit was utilized for DNA extraction (Qiagen Sciences INC, Germantown, MD, USA). WGS of isolates was performed on an Illumina MiSeq platform (Illumina, San Diego, CA, USA). Raw reads were cleaned using Trimmomatic v0.39 [89] to remove adapters and low-quality bases, followed by quality assessment with FastQC v0.11.9 [90]. De novo genome assembly was performed using Shovill v1.1.0, which employs SPAdes as the underlying assembler.v3.14.0 [91]. The quality of each assembly was evaluated with QUAST v5.0.2 [92], which provided standard metrics (total length, N50, number of contigs, GC content). Genome annotation was done with Prokka v1.14.6 [93]. The sequences were deposited in the Sequence Reads Archive (SRA) under BioProject PRJNA1405780, with BioSample ID: 1405780.

Species identification and strain typing were performed using KmerFinder v3.0.2 [94]. Sequence types (STs) were assigned via the PubMLST database [95], based on seven consensus genes (*dnaA*, *fusA*, *gyrB*, *leuS*, *pyrG*, *rlpB*, and *rpoB*), and plasmid replicon types were identified with PlasmidFinder v3.0 [96]. Antimicrobial resistance (AMR) genes, along with metal/biocide resistance and stress response determinants, were identified using AMRFinderPlus v4.2.5 [97] and ABRicate v1.0.1 [98] against the ResFinder database [99]. Class 1 integrons were detected with IntegronFinder v2.0.5 [100]. The virulome was analyzed using ABRicate v1.0.1 [98] against the Virulence Factors Database (VFDB) [101]. Genomic islands (GIs) were predicted with IslandViewer 4 [102]. Genome visualization was conducted using Proksee [93] to generate circular maps depicting the distribution of the main determinants identified. The outer membrane porins OmpC and OmpF were analyzed for sequence variations. Gene sequences were extracted from Prokka v1.14.6 (.gff) annotations and aligned using MAFFT v7.525 [103], with OmpC (accession no. KY086510.1) and OmpF (accession no. KY086519.1) as reference sequences.

To establish the phylogenetic context of our clinical *E. hormaechei* isolates within the Ecuadorian setting, a reference dataset was compiled comprising all 18 publicly available *E. hormaechei* whole-genome sequences (WGS) reported from Ecuador, retrieved from the NCBI GenBank database [104] (accessed 15 December 2025). The complete list of accession numbers is provided in Supplementary Materials, Table S1. The core genome phylogeny was inferred using Panaroo v1.5.2 [105] with the moderate cleaning mode, which identified a total of 3092 core genes shared among the genomes. These core genes were used to construct a phylogenetic tree using the maximum likelihood approach implemented in IQ-TREE v3.0.1 [106]. Tree robustness was assessed with 1000 ultrafast bootstrap replicates, and tree inference was further optimized using the integrated neural network option. For subtype classification of the strains, hsp60ECCtool [107] was used, which allowed the cluster, clade, and subspecies to be determined using the method of Sutton et al. [55]. The ribosomal MLST (rMLST) [108] was determined using the PubMLST database [95]. In cases where an exact rMLST profile match was not available, isolates were assigned to the closest matching rMLST profile based on sequence similarity. ENH_002 was most closely related to rST-63194, with a loci match of 52/53; ENH_003 was related to rST-179353, with a loci match of 49/53; El_PDT002917123 was related to rST-310084, with a loci match of 47/53; EL_PDT002917124 was related to rST-310084, with a loci match of 47/53; and, finally, EL_PDT002917126 was related to rST-310084, with a loci match of 47/53. In the context of MLST, the sequence types of ENH_003, EL_ASM4931666v1, and ECL_ASM4169912v1 were identified as novel. ENH_003 was found to be most closely aligned with ST 337, EL_ASM4931666v1 was most closely related to ST 1269,692, and ECL_ASM4169912v1 showed the closest resemblance to ST 1510,1962,3295,406. The Interactive Tree of Life (iTOL) v6 web platform [109] was used to visualize the phylogenetic tree.

## 5. Conclusions

This study describes the subspecies-level genomic features of *E. hormaechei* subsp. *xiangfangensis* and subsp. *hoffmannii* isolates recovered in Ecuador. The analyzed strains showed heterogeneous antimicrobial resistance profiles, including multidrug-resistant and largely susceptible phenotypes within the same healthcare setting. An ST136 isolate carried *bla*_CTX-M-15_ and *bla*_OXA-1_ together with additional resistance determinants associated with integron and plasmid sequences. Phylogenetic reconstruction situated these isolates within established subspecies and sequence type lineages and indicated relatedness to other Ecuadorian genomes. Together, these findings expand current knowledge of the genomic diversity of *E. hormaechei* in Ecuador and provide a foundation for future investigations into the population structure of the ECC.

## Figures and Tables

**Table 1:** Antibiotic susceptibility test (Kirby–Bauer Method).

		Diameter of Inhibition (cm ± SD)			
Antibiotic Class	Antibiotic Disks	ENH_002 *E. hormaechei* subsp. *hoffmannii*	ENH_003 *E. hormaechei* subsp. *xiangfangensis*	ENH_004 *E. hormaechei* subsp. *xiangfangensis*	ENH_007 *E. hormaechei* subsp. *xiangfangensis*
β-lactams	Ampicillin (10 μg)	0.73 ± 0.42 (R)	0.63 ± 0.35 (R)	0.57 ± 0.06 (R)	0.67 ± 0.06 (R)
	Amoxicillin/Clavulanate (20/10 μg)	0.57 ± 0.12 (R)	0.97 ± 0.25 (R)	0.63 ± 0.06 (R)	0.60 ± 0.17 (R)
	Piperacillin/Tazobactam (100/10 μg)	1.23 ± 0.1 (R)	1.60 ± 0.35 (R)	2.23 ± 0.40 (I)	1.83 ± 0.35 (R)
	Cefuroxime (30 μg)	1.20 ± 0.40 (R)	0.70 ± 0.10 (R)	0.73 ± 0.21 (R)	0.63 ± 0.15 (R)
	Cefotaxime (30 μg)	3.60 ± 0.36 (S)	0.83 ± 0.31 (R)	1.03 ± 0.15 (R)	0.63 ± 0.23 (R)
	Ceftazidime (30 μg)	3.23 ± 0.23 (S)	1.13 ± 0.15 (R)	1.63 ± 0.25 (R)	2.47 ± 0.49 (S)
	Cefepime (30 μg)	3.83 ± 0.78 (S)	2.57 ± 0.65 (S)	1.30 ± 0.50 (R)	1.83 ± 0.87 (R)
	Aztreonam (30 μg)	3.43 ± 0.40 (S)	1.57 ± 0.76 (R)	1.07 ± 0.49 (R)	1.13 ± 0.23 (R)
	Ertapenem (10 μg)	3.10 ± 0.26 (S)	1.60 ± 0.66 (R)	1.13 ± 0.49 (R)	1.57 ± 0.60 (R)
	Meropenem (10 μg)	3.47 ± 0.42 (S)	3.00 ± 0.56 (S)	2.17 ± 0.76 (I)	1.37 ± 0.55 (R)
	Imipenem (10 μg)	3.80 ± 0.89 (S)	2.97 ± 0.90 (S)	2.23 ± 0.64 (I)	2.20 ± 0.98 (I)
Aminoglycosides	Tobramycin (10 μg)	1.77 ± 0.12 (S)	1.30 ± 0.20 (I)	0.67 ± 0.12 (R)	0.63 ± 0.21 (R)
	Gentamicin (10 μg)	1.83 ± 0.06 (S)	1.63 ± 0.06 (I)	0.13 ± 0.15 (R)	0.10 ± 0.10 (R)
	Amikacin (30 μg)	2.40 ± 0.82 (S)	2.00 ± 0.10 (S)	1.93 ± 0.60 (I)	1.57 ± 0.12 (R)
Quinolones	Ciprofloxacin (5 μg)	2.37 ± 0.15 (S)	3.50 ± 0.17 (S)	0.33 ± 0.06 (R)	0.17 ± 0.12 (R)
	Levofloxacin (5 μg)	2.90 ± 0.90 (S)	2.93 ± 0.61 (S)	1.23 ± 0.31 (R)	2.53 ± 0.25 (S)
Tetracycline	Tetracycline (30 μg)	1.87 ± 0.06 (S)	0.17 ± 0.15 (R)	0.35 ± 0.07 (R)	0.30 ± 0.10 (R)
Chloramphenicol	Chloramphenicol (30 μg)	2.00 ± 0.10 (S)	1.43 ± 0.12 (I)	2.13 ± 0.07 (S)	2.13 ± 0.01 (S)
Folate Pathway Antagonists	Co-trimoxazole (25 μg)	2.27 ± 0.12 (S)	2.50 ± 0.10 (S)	0.10 ± 0.10 (R)	0.07 ± 0.06 (R)

R: Resistant; I: Intermediate; S: Susceptible.

**Table 2:** Genomic and epidemiological features of *E. hormaechei* isolates.

Features	Details			
	ENH_002 *E. hormaechei* subsp. *hoffmannii*	ENH_003 *E. hormaechei* subsp. *xiangfangensis*	ENH_004 *E. hormaechei* subsp. *xiangfangensis*	ENH_007 *E. hormaechei* subsp. *xiangfangensis*
Source	Clinical (community -acquired urine)	Clinical (surgical wound)	Clinical (traumatology wound)	Clinical (respiratory sample from ICU)
Genome size (bp)	4,766,956	4,720,818	4,650,111	4,649,924
No. of CDS	4519	4509	4345	4343
GC content	55.24%	54.81%	55.26%	55.26%
tRNA	87	84	77	80
rRNA	11	13	12	12
MLST	145	337	136	136
ARGs	-Aminoglycosides: *aadA4* -β-lactams: *bla_A_*_CT-14_ -Folate Antagonists: *sul1* -Fosfomycin: *fosA*	-β-lactams: *bla*_ACT-16_ -Chloramphenicol: *catA1* -Tetracycline: *tet(B)*	-Aminoglycosides: *aac(3)-IIa*; *aac(6′)-Ib-cr*; *aph(3″)-Ib*; *aph(6)-Id* -β-lactams: *bla*_ACT-16_; *bla*_CTX-M-15_; *bla*_OXA-1_; *bla*_TEM-1B_ -Chloramphenicol: *catB3* -Tetracycline: *tet(A)* -Folate Antagonists: *dfrA14*; *sul2* -Fosfomycin: *fosA* -Quinolones: *aac(6′)-Ib-cr*	-Aminoglycosides: *aac(3)-IIa*; *aac(6′)-Ib-cr*; *aph(3″)-Ib*; *aph(6)-Id* -β-lactams: *bla*_ACT-16_; *bla*_CTX-M-15_; *bla*_OXA-1_; *bla*_TEM-1B_ -Chloramphenicol: *catB3* -Tetracycline: *tet(A)* -Folate Antagonists: *dfrA14*; *sul2* -Fosfomycin: *fosA* -Quinolones: *aac(6′)-Ib-cr*
Virulence factors	-Nutrient Acquisition: *ent* (A, B, E, S); *fep* (G, C, A) -Regulation: *rpoS*, *phoP*, *rcsB*, *fur* -Secretion Systems: *vipA/tssB* -Immune Evasion: *gndA*; *galF* -Adhesion/Biofilm: *cgsG* -Invasion: *ompA*	-Nutrient Acquisition: *ent* (A, B, S); *fep* (D, G, C, A) -Regulation: *rpoS*, *phoP*, *rcsB*, *fur* -Immune Evasion: *gndA*; *galF* -Adhesion/Biofilm: *cgsG* -Invasion: *ompA*	-Nutrient Acquisition: *ent* (A, B, E, S); *fep* (D, G, C, A) -Regulation: *rpoS*, *phoP*, *rcsB*, *fur* -Secretion Systems: *vipB*/*tssC*; *hcp*/*tssD*; *tssF*; *vipA*/*tssB* -Immune Evasion: *gndA*; *galF* -Adhesion/Biofilm: *cgsG* -Invasion: *ompA*	-Nutrient Acquisition: *ent* (A, B, E, S); *fep* (D, G, C, A) -Regulation: *rpoS*, *phoP*, *rcsB*, *fur* -Secretion Systems: *vipB*/*tssC*; *hcp*/*tssD*; *tssF*; *vipA* -Immune Evasion: *gndA*; *galF* -Adhesion/Biofilm: *cgsG* -Invasion: *ompA*
SRGs	-Thermal: *clpK*; *kefB-GI*; *hsp20*; *shsP*; *trxLHR*; *psi-GI*; *yfdX1/X2*; *hdeD-GI* -Heavy metal: *pcoABCDERS*; *silABCEFPRS*; *terDWZ*	-Heavy metal: *terDWZ*; *arsC*	-Heavy metal: *pco*ABCDERS; *sil*ABCEFPRS)	-Heavy metal: *pco*ABCDERS; *sil*ABCEFPRS)
Integrons	Class 1 (*att*; *intI*; *qacE*Δ*1*)	n.d.	Class 1 (*intI*)	Class 1 (*intI*)
Plasmid content	IncFIA(HI1); IncFIB(pECLA); IncFII(pECLA); IncR	IncHI2; IncHI2A	n.d.	n.d.
Genomic Islands	42	45	34	36

ICU: intensive care unit; ARGs: antimicrobial resistance genes; SRGs: stress resistance genes; n.d.: not detected.

## Data Availability

The original contributions of this study are included in the article/Supplementary Materials and Supplementary Results. The whole-genome sequencing data for *E. hormaechei* will be deposited in the NCBI GenBank BioProject public archive under accession number PRJNA1405780, with biosample data available under accession numbers SAMN54756035 to SAMN54756038. The sequence data have also been made available at: https://drive.google.com/drive/folders/1N72OZqUkbbyFvRBROiuX5H3RPoMlfG-C?usp=sharing (accessed on 26 February 2026).

## Supplementary Materials

The following supporting information can be downloaded at: https://www.mdpi.com/article/10.3390/antibiotics15040387/s1. Supplementary Results. Raw data; Supplementary Results. Figure S1: Multiple sequence alignment of the Omp reference sequences and isolates ENH_003, ENH_004, and ENH_007; Supplementary Results. Figure S2: Main genomic features of *E. hormaechei* isolates; Supplementary Materials. Table S1: Accession numbers of the *E. hormaechei* genomes used for comparative analysis; Supplementary Results. Prophages and CRISPR Cas. Table S2. Prophages and the CRISPR-Cas System among the studied isolates. References [110,111] are cited in Supplementary Materials.
