# Supplementary material for: Whole-Genome Sequencing Reveals Heterogeneous Resistance Profiles and Selected Mobile Genetic Elements in Ecuadorian Clinical Enterobacter hormaechei subsp. xiangfangensis and subsp. hoffmannii
Source: Antibiotics (Basel). 2026 Apr 10;15(4):387. doi: 10.3390/antibiotics15040387 (PMC13113988; doi:10.3390/antibiotics15040387)
Supplement: Supplementary file 1 [file antibiotics-15-00387-s001.zip › Supplementary Results. Prophages and CRISPR Cas.pdf]

Supplementary Results. Prophages and CRISPR Cas

Putative phage-like regions were identified across the four isolates using PHASTER [1] and are summarized in the Table below. These regions showed sequence similarity to previously described bacteriophages, including *Enterobacter* phage ES18, filamentous phage SfI, mEp390, *Erwinia* phage vB_EhrS_59, *Erwinia* phage EtG, *Pseudomonas*-associated phage PPoW3, *Escherichia* phage HK639, and enterobacterial phage mEp237. However, given the use of draft genomes derived from short-read sequencing, these predictions should be interpreted cautiously, as genome fragmentation may affect the accurate delineation of phage boundaries and may result in incomplete or fragmented phage-like elements. In this context, no additional validation of phage completeness, integration signals, or taxonomic classification (e.g., using CheckV or alternative tools) was performed. Therefore, these regions are conservatively reported as putative phage-like sequences, and no assumptions are made regarding their integration status or lifestyle (e.g., temperate vs. lytic). Screening of these regions did not identify known antimicrobial resistance genes or virulence factors based on the databases used in this study; however, this observation should be interpreted with caution, as no dedicated analysis of viral contigs was conducted. Regarding CRISPR-Cas systems, bioinformatic analysis identified features consistent with a putative Type I system in all isolates, based on the detection of a *cas3* gene and a CRISPR array containing a single spacer (see Table below), using CRISPRCasFinder v4.3.2 [2]. However, the fragmented nature of the draft assemblies prevented reconstruction of complete *cas* operons and precise subtype classification. Given the repetitive structure of CRISPR loci and the limitations of short-read sequencing, the organization and completeness of these systems remain uncertain. Consequently, the functional status of the CRISPR-Cas systems could not be determined and should be interpreted as preliminary.

Table S2. ***Prophages and the CRISPR-Cas System*** **among the studied isolates**

**Features**	**Details**			
	**ENH_002**<br>***E. hormaechei*** **subsp.** ***hoffmannii***	**ENH_003**<br>***E. hormaechei*** **subsp.** ***xiangfangensis***	**ENH_004**<br>***E. hormaechei*** **subsp.** ***xiangfangensis***	**ENH_007**<br>***E. hormaechei*** **subsp.** ***xiangfangensis***
Prophage regions	PHAGE_Entero_SfI_NC_027339<br>PHAGE_Entero_ES18_NC_006949<br>PHAGE_ Entero_mEp390_NC_019721	PHAGE_Erwini_VB_EhrS_59_NC_048198<br>PHAGE_Entero_mEp390_NC_019721<br>PHAGE_Erwini_EtG_NC_047833<br>PHAGE Pseudo_PPoW 3 NC_023006	PHAGE Erwini VB EhrS 59 NC 048198<br>PHAGE_Entero_mEp390_NC_019721<br>PHAGE Escher HK639 NC 016158	PHAGE_Erwini_VB_EhrS_59_NC_048198<br>PHAGE_Entero_mEp390_NC_019721<br>PHAGE_Entero_mEp237_NC_019704
CRISPR-Cas	Type I (Class 1) (1 spacer)	Type I (Class 1) (1 spacer)	Type I (Class 1) (1 spacer)	Type I (Class 1) (1 spacer)

References

[1] Arndt, D.; Grant, J.R.; Marcu, A.; Sajed, T.; Pon, A.; Liang, Y.; Wishart, D.S. PHASTER: A better, faster version of the PHAST phage search tool. *Nucleic Acids Res.* 2016, *44*, W16–W21.

[2] Couvin, D.; Bernheim, A.; Toffano-Nioche, C.; Touchon, M.; Michalik, J.; Néron, B.; Rocha, E.P.C.; Vergnaud, G.; Gautheret, D.; Pourcel, C. CRISPRCasFinder, an update of CRISRFinder, includes a portable version, enhanced performance and integrates search for Cas proteins. *Nucleic Acids Res.* 2018, *46*, W246–W251. https://doi.org/10.1093/nar/gky425
