# Supplementary material for: Whole-Genome Sequencing Reveals Heterogeneous Resistance Profiles and Selected Mobile Genetic Elements in Ecuadorian Clinical Enterobacter hormaechei subsp. xiangfangensis and subsp. hoffmannii
Source: Antibiotics (Basel). 2026 Apr 10;15(4):387. doi: 10.3390/antibiotics15040387 (PMC13113988; doi:10.3390/antibiotics15040387)
Supplement: Supplementary file 1 [file antibiotics-15-00387-s001.zip › Supplemetary Materials. TableS1.pdf]

Table S1. Accession numbers of the *E. hormaechei* genomes used for comparative analysis.

#	Accession number	Genome assembly	rST	Province	Source	Species
1	ASM3001698v1	Enterobacter chengduensis genome assembly	221005	Guayas	Clinic	*E. chengduensis*
2	ASM4169912v1	Enterobacter cloacae genome assembly	293852	Esmeraldas	Enviroment	*E. cloacae*
3	ASM152602v1	Enterobacter hormaechei subsp. steigerwaltii genome assembly	71178	Pichincha	Clinic	*E. hormaechei*
4	ASM2996024v1	Enterobacter hormaechei genome assembly	56601	Pichincha	Clinic	*E. hormaechei*
5	ASM2996025v1	Enterobacter hormaechei genome assembly	221021	Pichincha	Clinic	*E. hormaechei*
6	ASM2996032v1	Enterobacter hormaechei genome assembly	179347	Pichincha	Clinic	*E. hormaechei*
7	ASM2996035v1	Enterobacter hormaechei genome assembly	56601	Pichincha	Clinic	*E. hormaechei*
8	ASM2996036v1	Enterobacter hormaechei genome assembly	179347	Pichincha	Clinic	*E. hormaechei*
9	ASM2996037v1	Enterobacter hormaechei genome assembly	179347	Pichincha	Clinic	*E. hormaechei*
10	ASM2017712v1	Enterobacter kobei genome assembly	147493	Pichincha	Enviroment	*E. kobei*
11	ASM2996046v1	Enterobacter kobei genome assembly	221022	Pichincha	Clinic	*E. kobei*
12	ASM4931660v1	Enterobacter ludwigii genome assembly	310082	Pichincha	Food	*E. ludwigii*
13	ASM4931664v1	Enterobacter ludwigii genome assembly	310083	Pichincha	Food	*E. ludwigii*
14	ASM4931666v1	Enterobacter ludwigii genome assembly	169890	Pichincha	Food	*E. ludwigii*
15	ASM4931668v1	Enterobacter ludwigii genome assembly	310084	Pichincha	Food	*E. ludwigii*
16	PDT002917123.1	Enterobacter ludwigii genome assembly	ND	Pichincha	Food	*E. ludwigii*
17	PDT002917124.1	Enterobacter ludwigii genome assembly	ND	Pichincha	Food	*E. ludwigii*
18	PDT002917126.1	Enterobacter ludwigii genome assembly	ND	Pichincha	Food	*E. ludwigii*
19	ENH_002 (this study)	Enterobacter hormaechei genome assembly	ND	Imbabura	Clinic	*E. hormaechei*
20	ENH_003 (this study)	Enterobacter hormaechei genome assembly	ND	Imbabura	Clinic	*E. hormaechei*

21	ENH_004 (this study)	Enterobacter hormaechei genome assembly	119008	Imbabura	Clinic	*E. hormaechei*
22	ENH_007 (this study)	Enterobacter hormaechei genome assembly	119008	Imbabura	Clinic	*E. hormaechei*
