# Supplementary material for: Whole-Genome Sequencing Reveals Heterogeneous Resistance Profiles and Selected Mobile Genetic Elements in Ecuadorian Clinical Enterobacter hormaechei subsp. xiangfangensis and subsp. hoffmannii
Source: Antibiotics (Basel). 2026 Apr 10;15(4):387. doi: 10.3390/antibiotics15040387 (PMC13113988; doi:10.3390/antibiotics15040387)
Supplement: Supplementary file 1 [file antibiotics-15-00387-s001.zip › Supplemetary Results. Figure S1. Figure S2.pdf]

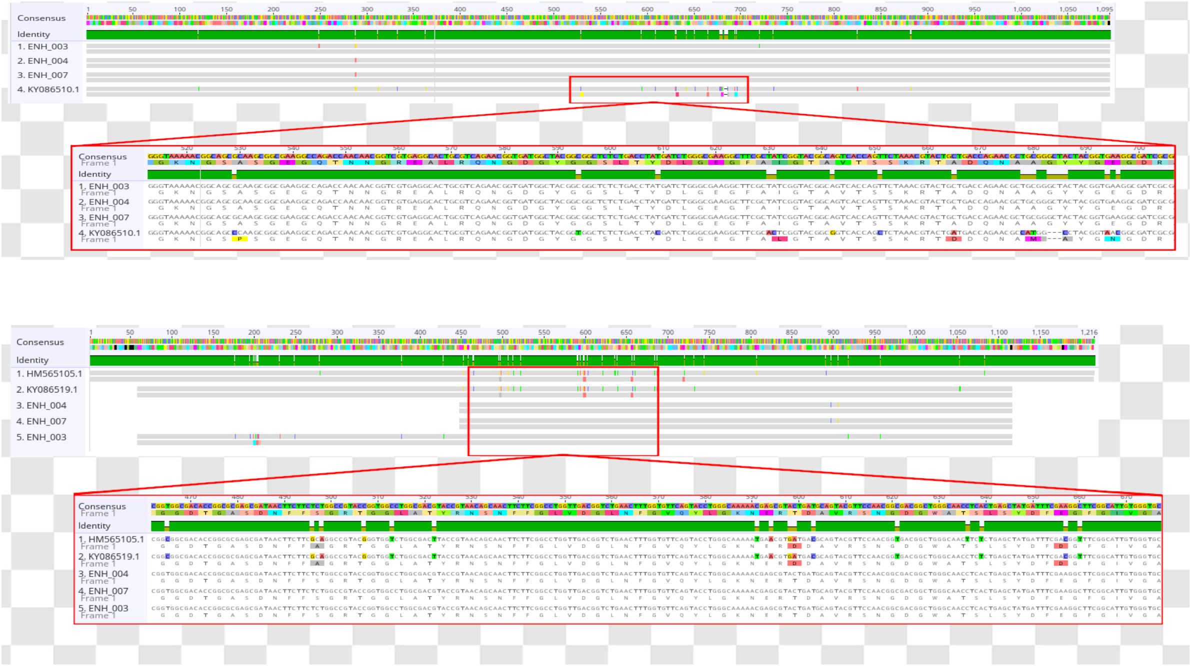

Figure S1. Multiple sequence alignment of the Omp reference sequences and isolates ENH_003, ENH_004, and ENH_007. (Upper panel). Alignment of the OmpC reference sequence ((accession code: KY086510.1)). (Lower panel). Alignment of the OmpF reference sequence (accession code: KY086519.1). The region containing nucleotide substitutions associated with nonsynonymous amino acid changes is shown in a magnified panel. Nucleotide and corresponding amino acid variations are highlighted in color to facilitate visualization of sequence differences and their predicted impact on the protein sequence.

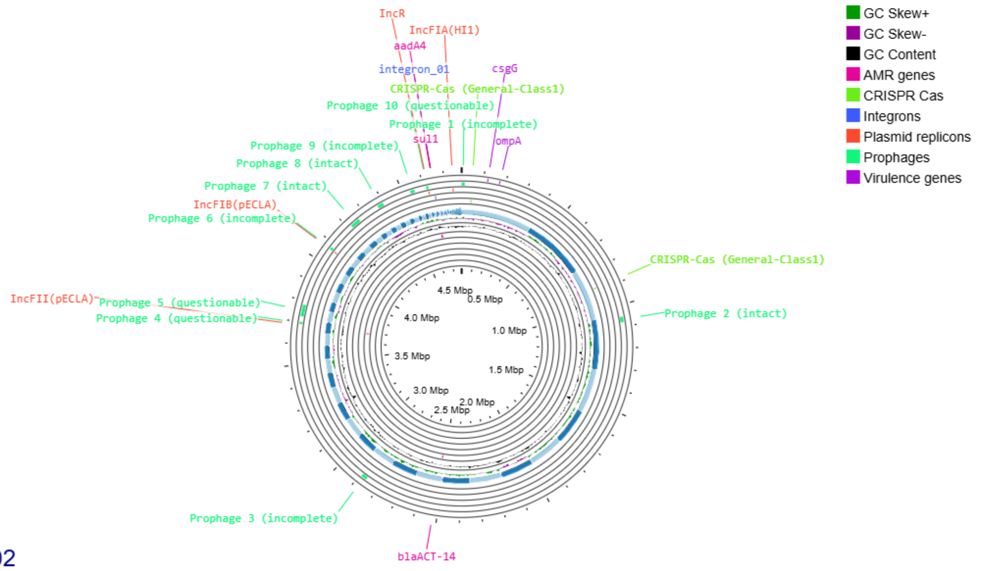

ENH_002

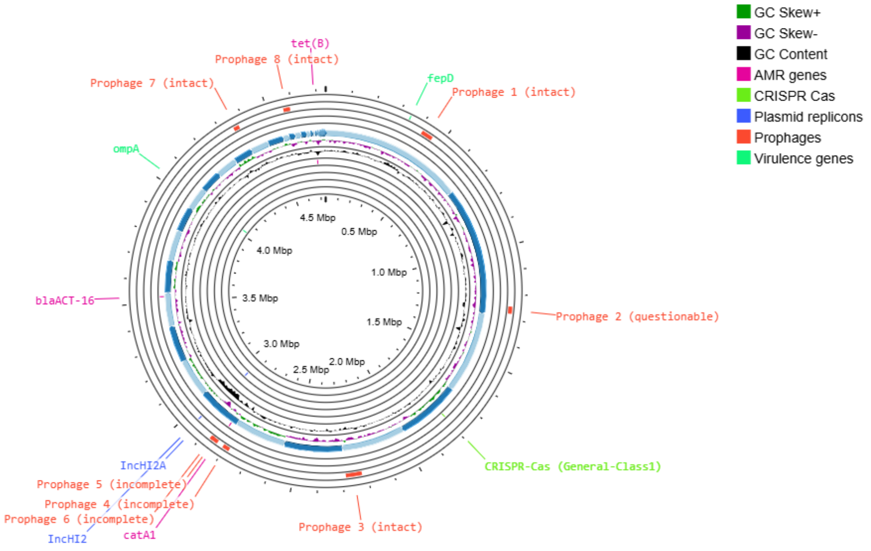

ENH_003

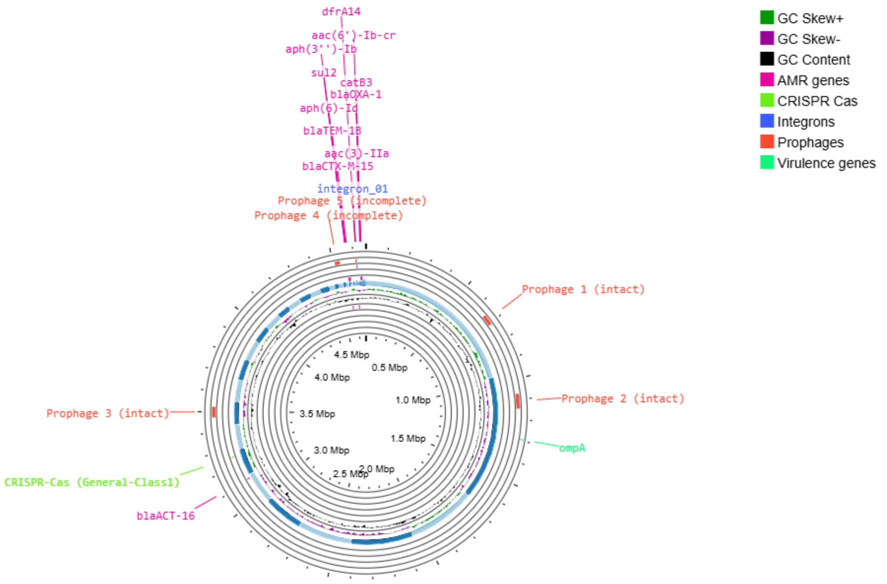

ENH_004

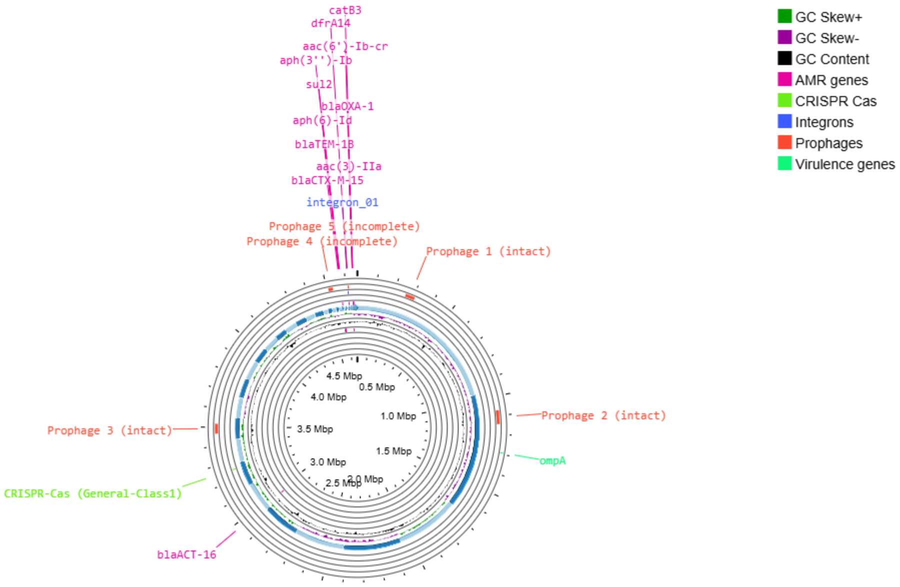

ENH_007

Figure S2. Main genomic features of *E. hormaechei* isolates. Circular maps of ENH_002, ENH_003, ENH_004, and ENH_007.
